# Galanin and glypican-4 levels depending on metabolic and cardiovascular risk factors in patients with polycystic ovary syndrome

**DOI:** 10.20945/2359-3997000000340

**Published:** 2021-03-19

**Authors:** Sunduz Ozlem Altinkaya

**Affiliations:** 1 Adnan Menderes University Faculty of Medicine Aydin Turkey Adnan Menderes University, Faculty of Medicine, Aydin, Turkey.

**Keywords:** Polycystic ovary syndrome, galanin, glypican-4

## Abstract

**Objective:**

Galanin is a neuropeptide which has effects not only on metabolic syndrome but also on reproduction. Glypican-4 is an adipokine associated with insulin sensitivity by interacting directly with the insulin receptor. This study evaluated serum concentrations of galanin and glypican-4 in relation with the hormonal profile as well as metabolic and cardiovascular risk factors in patients with and without polycystic ovary syndrome (PCOS).

**Subjects and methods:**

A total of 44 women with PCOS and 44 age-matched controls were eligible. Hirsutism scores, hormonal profile, metabolic and cardiovascular risk factors as well as galanin and glypican-4 levels were evaluated in each subject.

**Results:**

Women with PCOS exhibited lower levels of galanin (20.2 pg/mL versus 26.4 pg/mL, p = 0.002) and higher concentrations of glypican-4 (3.1 ng/mL versus 2.6 ng/mL, p < 0.001) than controls. Both adipokines were correlated positively with body mass index (BMI), insulin, triglyceride and Homeostasis Model Assessment (HOMA) index; glypican-4 also showed positive correlations with fasting blood glucose, free testosterone, modified Ferriman-Gallwey scores (p < 0.05). Multiple Linear Regression analyses showed that PCOS and BMI were the best predictors affecting galanin levels with a decreasing and increasing effect respectively; however BMI was the best predictor affecting glypican-4 levels with an increasing effect (p < 0.001).

**Conclusion:**

Galanin levels were lower and glypican-4 levels were higher in women with PCOS than controls. Further studies are needed to determine whether these adipokines could be used as additional markers for insulin sensitivity and lipid profile and whether they might play a role in the pathogenesis of PCOS, in which metabolic cardiovascular risks are increased.

## INTRODUCTION

Polycystic ovary syndrome (PCOS) is the most common endocrine disorder and the cause of ovulatory dysfunction in women of reproductive age. It occurs at a frequency of 5–15%, depending on the diagnostic criteria applied ( 1 , 2 ). In spite of the variable clinical expression, the syndrome is classically described by ovulatory dysfunction, hyperandrogenism and polycystic ovaries ( 1 ). Unfortunately, the multisystem effects of the syndrome span from systemic metabolic disturbances to reproductive dysfunction, in addition to long-term cardiovascular event and cancer risk ( 1 , 2 ). The more severe PCOS phenotypes are associated with further emphasis of cardiovascular disease risk ( 3 ). Both obese and lean women with PCOS have various levels of insulin resistance. Given the interrelation with insulin resistance, all women diagnosed with PCOS need to be evaluated for the risk of metabolic syndrome and associated diseases, such as type 2 diabetes, hypertension, hyperlipidaemia, and the possible risk of some clinical emergency events, including acute myocardial infarction and stroke ( 4 ).

Galanin, a member of the galanin peptide family (galanin, galanin-like peptide, alarin), is a 29-amino-acid neuropeptide, and involved in regulating appetite, obesity, insulin resistance, hypertension and metabolism ( 5 ). It was first isolated from porcine intestine by Tatemoto and cols. ( 6 ) nearly 35 years ago. This peptide is not only found in central and peripheral nervous systems but also in the human carotid body, skeletal and heart muscle, adipose tissue and pancreas ( 7 , 8 ). Galanin has effects on metabolic syndrome with regard to food consumption, preference for a high-fat diet, elevation in the probability of obesity and dyslipidaemia and decreased insulin resistance and blood pressure to relieve the risk for type 2 diabetes mellitus and hypertension ( 5 ). Galanin is a crucial neuropeptide which supports glucose transport via GLUT-4, an insulin-regulated glucose transporter. The overall effect of galanin on the metabolic syndrome may be summarised briefly into two categories. First, it causes an increase in GLUT-4 translocation to promote glucose intake, therefore elevating insulin sensitivity. Second, it decreases insulin secretion from pancreatic islet cells ( 5 ). It may interact with other peptides that play roles in regulating appetite, such as neuropeptide-Y and leptin, and this orchestration could play a role in the pathogenesis of the metabolic syndrome ( 9 ). In addition, galanin is a target agent for sex steroids, serving as molecular motifs integrating the control of metabolism as well as reproduction ( 10 ). It is released in a pulsatile manner, similar to the gonadotrophin-releasing hormone (GnRH) ( 10 ), and stimulates the secretion of luteinizing hormone (LH) in the porcine; in mice, it inhibits LH secretion ( 11 , 12 ).

Glypican-4 was first discovered in 1995 in the kidneys and developing brain tissue of mice by Watanabe and cols. ( 13 ). Gesta and cols. ( 14 ) demonstrated that the genetic expression of glypican-4 differs in visceral and subcutaneous fat tissues. The authors found that lower subcutaneous and higher visceral adipose tissue expression of glypican-4 appear to be associated with an increased waist-hip ratio (WHR) and body mass index (BMI), indicating higher risks for metabolic and cardiovascular complications ( 14 ). Recently, glypican-4 has been identified as a novel adipokine, which is a cell surface proteoglycan and enhances insulin receptor signalling and adipocyte differentiation by interacting directly with the insulin receptor, unlike other insulin sensitisers ( 15 ). The binding of glypican-4 to the insulin receptor regulates insulin activation and downstream signalling as an insulin sensitiser. Furthermore, circulating glypican-4 levels are higher in subjects with impaired glucose tolerance and positively correlated with BMI and WHR, the Homeostasis Model Assessment (HOMA) index ( 16 ).

Galanin and glypican-4 are likely to be main regulators in glucose and lipid metabolism and might be associated with insulin resistance and obesity, and PCOS is related with insulin resistance and other metabolic disorders such as dyslipidaemia, hypertension, endothelial dysfunction with reduced vascular compliance and, consequently atherosclerosis. In this context, it was analysed whether there was an alteration in serum galanin and glypican-4 levels in a group of women with and without PCOS. This study also set out to correlate both adipokine levels in relation with hormonal and metabolic profiles as well as cardiovascular risk factors to investigate the associations between some markers in connection with cardiovascular disease.

## SUBJECTS AND METHODS

The design of the present study was approved by the Ethical Committee and Institutional Review Board of the Adnan Menderes University Faculty of Medicine, in accordance with the Declaration of Helsinki of the World Medical Association. After providing verbal information written informed consents were obtained from all women participating in the study.

This study applied G power analysis to calculate the sample size. Based on serum levels of galanin in the study by Bidzińska-Speichert and cols. ( 17 ) and on serum levels of glypican-4 in the study by Jędrzejuk and cols. ( 18 ), when the effect size for galanin (pg/mL) and glypican-4 (ng/mL) was 1.298 and 1.119, respectively; α = 0.05 was the two-sided hypothesis, statistical power = 90%, a minimum of 17 subjects for galanin and 20 subjects for glypican-4 in each group should be studied. More patients were reached to increase the power of the study. A total of 44 women with PCOS and 44 age-matched controls were eligible. The Rotterdam criteria were applied for the diagnosis of PCOS ( 19 ) in the presence of at least two of the following: 1) oligomenorrhea and/or anovulation 2) biochemical and/or clinical hyperandrogenism 3) ultrasonographic appearance of polycystic ovaries (PCO) (multiple cysts > 12 in number of 2-9 mm size), along with the exclusion of other hormonal, metabolic and cardiovascular aetiologies (congenital adrenal hyperplasia, virilising ovarian or adrenal tumour, Cushing syndrome, hyperprolactinemia, diabetes mellitus, elevated blood pressure and any other cardiovascular diseases). No subject smoked or, consumed alcohol. Over 3 months preceding the study, none of the participants had been on hormonal contraceptives and any other medications or a diet which might affect lipid and carbohydrate metabolism. As controls, 44 age-matched healthy women, who had regular menses with no clinical and/or biochemical hyperandrogenism, were eligible.

A detailed clinical history was obtained, and physical examination was performed for all voluntary women that participated in the present study. The BMI was calculated in kg/m^2^ unit. Routine laboratory measurements were performed as follows: carbohydrate metabolism parameters including fasting blood glucose, fasting insulin, lipid profile including triglycerides (TG), total cholesterol, low-density lipoprotein (LDL), high-density lipoprotein (HDL), pituitary hormones including follicle-stimulating hormone (FSH) and luteinising hormone (LH), sex steroids including oestradiol (E2), dehydroepiandrosterone sulphate (DHEA-SO4) and free testosterone (fT) and thyroid hormones including thyroid-stimulating hormone (TSH), free thyroxine levels (free T3 and free T4). Insulin resistance (IR) was determined by the Homeostasis Model Assessment (HOMA) index (fasting glucose (mg/dl) x fasting insulin (μU/mL)/405) ( 20 ). Phenotypical classification was performed as described previously ( 21 ). All sampling procedures were performed in the early follicular phase (day 2-5 of the menstrual cycle) in the morning after an overnight fast. In oligomenorrheic women samples were taken in their spontaneous cycle (day 2-5). Among the 44 patients with PCOS, only 2 had amenorrhea, in whom, progesterone withdrawal bleeding was achieved with medroxyprogesterone acetate 2*5 mg for 6 days (Tarlusal^®^, Deva Holding, Istanbul). Circulating hormone levels were measured in an auto-analyser (C8000 Architect, Abbott, Abbott Park, IL, USA) using the Chemiluminescent Microparticle Immunoassay (CMIA) method with Architect hormone kits. Serum galanin and glypican-4 levels were assessed by an enzyme-linked immunosorbent assay (Human GAL (Galanin) ELISA Kit, China). The intra- and inter-assay coefficients of variations for both adipokines were < 10%. The detection ranges for galanin and glypican-4 were 15.625-1000 pg/mL and 0.156-10 ng/mL, respectively.

Data analysis was performed using the software IBM SPSS Statistics version 17.0 (IBM Corporation, Armonk, NY, USA). Whether the distributions of continuous variables were normal or not was determined by the Kolmogorov Smirnov test. Data were expressed as mean ± SD or median (interquartile range), where applicable. The mean differences between control and PCOS groups were compared by Student's t test; otherwise, Mann Whitney's U test was applied for comparisons of the not normally distributed data. Degrees of association between continuous variables were evaluated by Spearman's Rank Correlation analyses. Whether the differences in galanin and glypican-4 levels between control and PCOS groups were keeping on or not was evaluated by Multiple Linear Regression Analyses after adjustment for all possible confounding factors. Any variable whose univariable test had a p value < 0.10 was accepted as a candidate for the multivariable model, along with all variables of known clinical importance. The coefficient of regression, 95% confidence interval and t-statistic for each independent variable were also calculated. Because they were not normally distributed, logarithmic transformation was used for both galanin and glypican-4 measurements in regression analyses. A p value less than 0.05 was considered statistically significant. However, for all possible multiple comparisons, Bonferroni Correction was applied for controlling Type I error. When Bonferroni adjustment was applied, a p value less than 0.025 was significant.

## RESULTS

**Figure 1:**
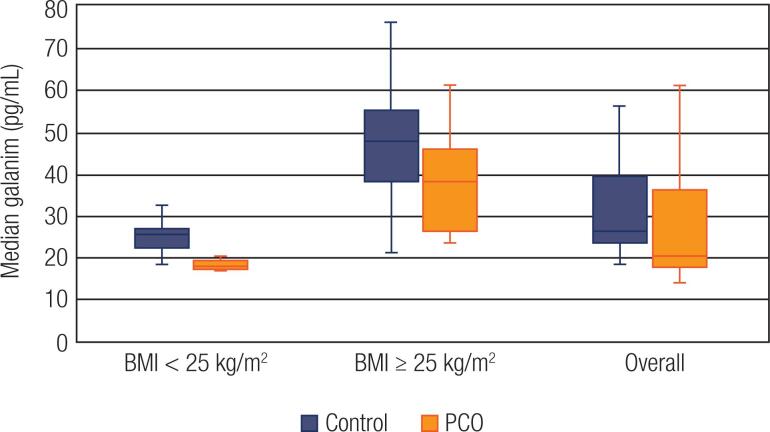
Galanin levels in PCOS and controls regarding BMI. The horizontal lines in the middle of each box indicates the median galanin levels, while the top and bottom borders of the box mark the 25^th^ and 75^th^ percentiles, respectively. The whiskers above and below the box mark indicates the maximum and minimum levels, respectively.

**Figure 2:**
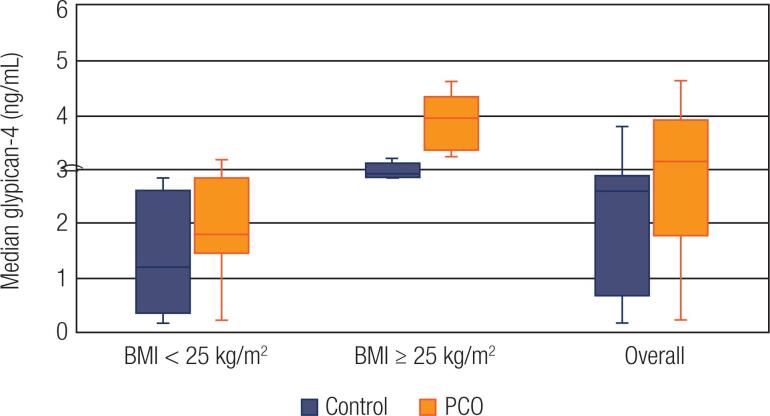
Glypican-4 levels in PCOS and controls regarding BMI. The horizontal lines in the middle of each box indicates the median glypican-4 levels, while the top and bottom borders of the box mark the 25^th^ and 75^th^ percentiles, respectively. The whiskers above and below the box mark indicates the maximum and minimum levels, respectively.

A total of 44 women with PCOS and 44 controls voluntarily participated. Among the 44 cases of PCOS, 29 (65.9%) women were phenotype A, 4 (9.1%) women were phenotype B, 5 (11.4%) women were phenotype C, and 6 (13.6%) women were phenotype D. The median galanin levels were lower (20.2 pg/mL vs. 26.4 pg/ mL, p = 0.002), whereas the median glypican-4 levels were higher (3.1 ng/mL vs. 2.6 ng/mL) in women with PCOS as compared to the women in the control group. Subgroup analyses regarding the BMI were also performed. Participants with BMI < 25 kg/m^2^ were classified as lean, whereas women with a BMI ≥ 25 kg/m^2^, comprised the overweight/obese group. Subgroup analysis indicated that the median galanin concentrations were lower in lean patients with PCOS (18 pg/mL vs. 23.3 pg/mL, p < 0.001) compared to controls. The PCOS patients with BMI ≥ 25 kg/m^2^ also exhibited lower galanin concentrations, although this difference was not significant (38.4 pg/mL vs. 47.9 pg/mL, p = 0.043) after Bonferroni correction was applied. The galanin measurements of overweight and obese women were higher than those of lean women in both PCOS and control groups (38.4 pg/mL vs 18 pg/mL, p < 0.001 and 47.9 pg/mL and 23.3 pg/mL, p < 0.001, respectively). ( Table 1 , Figure 1 ). The glypican-4 levels were similar between PCOS and controls in the lean group (1.8 ng/mL vs. 1.2 ng/mL, p = 0.124), whereas overweight/obese PCOS women showed higher levels than the BMI- matched controls (3.9 ng/mL vs. 2.9 ng/mL, p < 0.001). When PCOS and control groups were evaluated on their own, the glypican-4 levels were lower in lean women in both groups (1.8 ng/mL vs. 3.9 ng/mL, p < 0.001 and 1.2 ng/mL vs. 2.9 ng/mL, p < 0.001) ( Table 1 , Figure 2 ). The demographic, clinical, biochemical, hormonal and metabolic characteristics of women with PCOS and controls with regard to BMI are summarised in Table 1 .

**Table 1:** Demographic, clinical, biochemical and hormonal characteristics of women with PCOS and controls regarding BMI

	PCOS			Controls			pa	pb	pc	pd	pe
	BMI<25 (n=24)	BMI≥25 (n=20)	Total (n=44)	BMI<25 (n=29)	BMI≥25 (n=15)	Total (n=44)					
Age (years)	23.4±5.2	23.5±5.7	23.5±5.4	23.8±4.8	27.1±6.7	24.9±5.6	0.227 †	0.786 †	0.103 †	0.936 †	0.106 †
BMI (kg/m^2^)			25.2±3.8			22.8±2.4	**<0.001** †				
FBG (mg/dL)	90.7±11	92.3±9.5	91.4±10.3	87.6±5.8	89.1±4.1	88.1±5.3	0.062 †	0.220 †	0.196 †	0.615 †	0.363 †
FI (μIU/mL)	9.3(6.7)	12.8(9.9)	10.5 (8.8)	8(3.1)	10.1(6.5)	8.1(4)	**0.002** ‡	0.174 ‡	**0.013** ‡	**0.009** ‡	0.173 ‡
HOMA-IR	2(1.4)	3(2.3)	2.4 (1.9)	1.7(0.8)	2.1(1.3)	1.8(0.9)	**<0.001** ‡	0.136 ‡	**0.009** ‡	**0.012** ‡	0.110 ‡
TC (mg/dL)	183(46)	179(37.7)	180(45.2)	164(34.5)	186(39)	165.5(33.7)	0.108 ‡	0.088 ‡	0.882 ‡	0.944 ‡	0.162 ‡
HDL(mg/dL)	52.2±11.9	45.1±12.9	49±12.7	54.3±10.1	53.2±9.5	53.9±9.8	**0.045** †	0.498 †	0.049 †	0.064 †	0.735 †
LDL(mg/dL)	113.6±20.8	112.8±29.6	113.3±24.9	97±19	109.3±25.6	101.2±22	**0.018** †	**0.004** †	0.710 †	0.920 †	0.079 †
TG (mg/dL)	92.5(59.5)	119(104.5)	95.5(62)	77(31)	93(45)	80.5(41.2)	0.027‡	0.153 ‡	0.149 ‡	0.075 ‡	0.116 ‡
fT(pg/mL)	2.6(1)	4(1.1)	3(1.9)	1.6(1.2)	1.1(0.8)	1.2(1.1)	**<0.001** ‡	**<0.001** ‡	**<0.001** ‡	**<0.001** ‡	**0.023** ‡
DHEA-SO4 (μg/dL)	300.5(220.7)	411(268.5)	330(244.2)	272(127.5)	241(177)	258.5(128.2)	**0.002** ‡	0.155 ‡	**0.002** ‡	0.487 ‡	0.081 ‡
fT3 (pg/mL)	3.2±0.4	3.1±0.6	3.1±0.5	3.1±0.5	2.9±0.3	3±0.4	0.221 †	0.347 †	0.358 †	0.584 †	0.418 †
fT4 (pg/mL)	1.1(0.3)	1.1(0.2)	1.1(0.2)	1.1(0.2)	1.1(0.2)	1.1(0.2)	0.350 ‡	0.537 ‡	0.419 ‡	0.654 ‡	0.496 ‡
TSH (μIU/mL)	1.6(1.5)	1.7(1.5)	1.7(1.5)	1.8(1.1)	1.6(0.8)	1.7(1)	0.917 ‡	0.830 ‡	0.633 ‡	0.715 ‡	0.683 ‡
FSH (mIU/mL)	4.2(1.2)	3.9(1.6)	4.2(1.2)	4.7(1.6)	4.5(1.2)	4.7(1.4)	**0.017** ‡	0.062 ‡	0.191 ‡	0.311 ‡	0.552 ‡
LH (mIU/mL)	7.6(9.4)	7(7.2)	7.2(8)	4.8(2.5)	5(1.5)	4.8(1.9)	**<0.001** ‡	**<0.001** ‡	**0.011** ‡	0.494 ‡	0.990 ‡
E2 (pg/mL)	47.5(43.5)	46(39.8)	47.5(38)	65(32.5)	70(48)	65(37.2)	**0.047** ‡	0.198 ‡	0.107 ‡	0.680 ‡	0.594 ‡
PRL (mIU/mL)	15±3	15.9±5.2	15.4±4.1	16.1±4.4	15.1±4.1	15.7±4.3	0.727 †	0.306 †	0.617 †	0.484 †	0.492 †
mFG	9(6.7)	11(3)	10(5.2)	3(2)	4(1)	3(1)	**0.047** ‡	**<0.001** ‡	**<0.001** ‡	**<0.001** ‡	*<0.001* ‡
Galanin (pg/mL)	18(2)	38.4(19.9)	20.2(18.8)	23.3(4.3)	47.9(17)	26.4(16.2)	**0.002** ‡	**<0.001** ‡	0.043 ‡	**<0.001** ‡	**<0.001** ‡
Glypican-4 (ng/mL)	1.8(1.4)	3.9(1)	3.1(2.1)	1.2(2.3)	2.9(0.2)	2.6(2.2)	**<0.001** ‡	0.124 ‡	**<0.001** ‡	**<0.001** ‡	**<0.001** ‡

BMI: body mass index; FBG: fasting blood glucose; FI: fasting insulin; HOMA-IR: Homeostasis Model Assessment-Insulin resistance; TC: total cholesterol; HDL: high density lipoprotein; LDL: low density lipoprotein; TG: triglycerides; fT: free testosterone; DHEA-SO4: dehydroepiandrosterone sulphate; TSH: thyroid stimulating hormone; FSH: follicle stimulating hormone; LH: luteinizing hormone; E2: estradiol; PRL: prolactin; mFG: modified Ferriman–Gallwey.

pa difference between PCOS and controls.

pb difference between PCOS and controls BM I <25, p value less than 0.025 was considered statistically significant with regard to Bonferroni adjustment.

pc difference between PCOS and controls BMI ≥25, p value less than 0.025 was considered statistically significant with regard to Bonferroni adjustment.

pd difference between BMI <25 and BMI ≥25 subgroups in patients with PCOS, p value less than 0.025 was considered statistically significant with regard to Bonferroni adjustment.

pe difference between BMI <25 and BMI ≥25 subgroups in controls, p value less than 0.025 was considered statistically significant with regard to Bonferroni adjustment.

† Student's *t* test.

‡ Mann Whitney U test.

Circulating galanin concentrations were positively correlated with BMI, fasting insulin, triglyceride, glypican-4 levels and HOMA-IR and negatively correlated with LH levels when all participants were evaluated (p < 0.05). In the PCOS group, galanin showed a positive correlation with BMI, fasting insulin, triglyceride, free testosterone and glypican-4 levels, HOMA-IR and mFG scores; the correlation was negative with HDL levels. In controls, only BMI, mFG scores and glypican-4 levels were positively correlated with galanin levels (p < 0.025 according to the Bonferroni correction). Glypican-4 levels were positively correlated with BMI, fasting blood glucose, fasting insulin, triglyceride, free testosterone and galanin levels as well as HOMA-IR and mFG scores and negatively correlated with HDL and FSH levels in the total patient group (p < 0.05). In the PCOS group, glypican-4 levels were positively correlated with BMI, fasting insulin, triglyceride, free testosterone and, galanin levels, HOMA-IR and mFG scores. In controls, glypican-4 levels showed a positive correlation with galanin levels as well as age, BMI and mFG scores, whereas DHEA-SO4 and free testosterone levels were negatively correlated (p < 0.025 according to the Bonferroni correction). Tables 2 and 3 demonstrate the correlations of both galanin and glypican-4 levels with clinical, metabolic, hormonal and androgen excess parameters.

**Table 2:** Correlations between galanin levels and clinical, metabolic, hormonal and androgen excess parameters

	All patients (n=88)		PCOS (n=44)		Controls (n=44)	
	r	p a †	r	p b †	r	p b †
Age (years)	0.087	0.422	-0.028	0.858	0.149	0.335
BMI (kg/m^2^)	0.689	**<0.001**	0.966	**<0.001**	0.768	**<0.001**
FPG (mg/dL)	0.017	0.872	0.100	0.519	0.108	0.484
FI (μIU/mL)	0.216	**0.043**	0.488	**<0.001**	0.199	0.196
HOMA-IR	0.211	**0.048**	0.474	**<0.001**	0.217	0.157
TC (mg/dL)	0.025	0.819	-0.094	0.546	0.252	0.099
HDL(mg/dL)	-0.192	0.072	-0.467	**<0.001**	-0.009	0.953
LDL(mg/dL)	-0.063	0.557	-0.114	0.463	0.187	0.224
TG(mg/dL)	0.230	**0.031**	0.358	**<0.001**	0.284	0.062
fT(pg/mL)	-0.007	0.946	0.652	**<0.001**	-0.124	0.423
DHEA-SO4 (μg/dL)	-0.069	0.520	0.170	0.269	-0.076	0.622
fT3 (pg/mL)	-0.102	0.344	0.052	0.738	-0.174	0.260
fT4 (pg/mL)	-0.155	0.151	-0.032	0.836	-0.175	0.255
TSH (μIU/mL)	-0.017	0.873	0.104	0.503	-0.204	0.185
FSH (μIU/mL)	-0.111	0.302	-0.149	0.336	-0.325	0.031
LH (μIU/mL)	-0.222	**0.038**	-0.044	0.776	-0.085	0.584
E2 (pg/mL)	0.089	0.410	-0.101	0.513	0.162	0.295
PRL (μIU/mL)	0.122	0.259	0.200	0.192	0.017	0.913
mFG	0.202	0.059	0.693	**<0.001**	0.407	**0.006**
Glypican4 (ng/mL)	0.651	**<0.001**	0.965	**<0.001**	0.770	**<0.001**

BMI: body mass index; FPG: fasting plasma glucose; FI: fasting insulin; HOMA-IR: Homeostasis Model Assessment-Insulin resistance; TC: total cholesterol; HDL: high density lipoprotein; LDL: low density lipoprotein; TG: triglycerides; fT: free testosterone; DHEA-SO4: dehydroepiandrosterone sulphate; fT3-fT4: free thyroxine levels; TSH: thyroid stimulating hormone; FSH: follicle stimulating hormone; LH: luteinizing hormone; E2:estradiol; PRL: prolactin; mFG: modified Ferriman–Gallwey.

a p value less than 0.05 was considered statistically significant;

b p value less than 0.025 was considered statistically significant with regard to Bonferroni adjustment.

r: Correlation coefficient;

† Spearman's Rank Correlation test.

**Table 3:** Correlations between glypican-4 levels and clinical, metabolic, hormonal and androgen excess parameters

	All patients (n=88)		PCOS (n=44)		Controls (n=44)	
	r	p a †	r	p b †	r	p b †
Age (years)	0.175	0.104	0.057	0.713	0.434	**0.003**
BMI (kg/m^2^)	0.971	**<0.001**	0.999	**<0.001**	0.999	**<0.001**
FPG (mg/dL)	0.230	**0.031**	0.155	0.314	0.242	0.114
FI (μIU/mL)	0.433	**<0.001**	0.463	**0.002**	0.231	0.131
HOMA-IR	0.457	**<0.001**	0.455	**0.002**	0.281	0.064
TC (mg/dL)	0.095	0.377	-0.110	0.477	0.302	0.046
HDL (mg/dL)	-0.334	**<0.001**	-0.508	**<0.001**	0.086	0.581
LDL (mg/dL)	0.099	0.358	-0.120	0.436	0.270	0.077
TG (mg/dL)	0.292	**0.006**	0.366	**0.015**	0.111	0.475
fT (pg/mL)	0.348	**<0.001**	0.631	**<0.001**	-0.339	**0.024**
DHEA-SO4	0.036	0.736	0.133	0.388	-0.363	**0.015**
fT3 (pg/mL)	0.027	0.800	0.101	0.515	-0.263	0.085
fT4 (pg/mL)	-0.065	0.546	-0.065	0.673	-0.154	0.317
TSH (μIU/mL)	0.012	0.911	0.121	0.433	-0.126	0.414
FSH (μIU/mL)	-0.228	**0.033**	-0.178	0.247	-0.132	0.393
LH (μIU/mL)	0.085	0.433	-0.049	0.753	-0.140	0.365
E2 (pg/mL)	-0.164	0.127	-0.157	0.310	0.067	0.664
PRL (μIU/mL)	0.055	0.613	0.147	0.340	-0.088	0.569
mFG	0.645	**<0.001**	0.668	**<0.001**	0.526	**<0.001**
Galanin (pg/mL)	0.651	**<0.001**	0.965	**<0.001**	0.770	**<0.001**

BMI: body mass index; FPG: fasting plasma glucose; FI: fasting insulin; HOMA-IR: Homeostasis Model Assessment-Insulin resistance; TC: total cholesterol; HDL: high density lipoprotein; LDL: low density lipoprotein; TG: triglycerides; fT: free testosterone; DHEA-SO4: dehydroepiandrosterone sulphate; fT3-fT4: free thyroxine levels; TSH: thyroid stimulating hormone; FSH: follicle stimulating hormone; LH: luteinizing hormone; E2: estradiol; PRL: prolactin; mFG: modified Ferriman–Gallwey.

a p value less than 0.05 was considered statistically significant;

b p value less than 0.025 was considered statistically significant with regard to Bonferroni adjustment.

r: Correlation coefficient;

† Spearman's Rank Correlation test.

The best predictor(s) which effect both galanin and glypican-4 concentrations were also evaluated by Multiple Linear Regression analyses, after adjustment for all possible confounding factors. As a result of univariate statistical analysis, variables with p < 0.01 were included in the linear regression model as candidate risk factors. Since galanin and glypican-4 levels were distributed far from normal, logarithmic transformation for both measurements was performed in the regression analysis. As there was a functional connection between fasting blood glucose and insulin levels and HOMA-IR, only HOMA-IR was included in the regression model instead of fasting blood glucose and insulin. Multiple Linear Regression analyses showed that PCOS and BMI appear to be independent risk factors affecting galanin levels (p < 0.001). When the correction was made according to other factors, the effect of being in the PCOS group compared to the control group continued to decrease the level of galanin (B = −0.472, 95%CI: −0.577 to −0.368 and p < 0.001). In addition, as the BMI increased, galanin levels continued to increase independently of other factors (B = 0.113, 95%CI: 0.097-0.129 and p < 0.001). Regarding the glypican-4 levels, the BMI was an independent risk factor (p < 0.001), and the significant effect of PCOS on glypican-4 levels in the univariate analysis disappeared in Multiple Linear Regression analyses (p = 0.181). As the BMI increased, the glypican-4 levels continued to increase independently of other factors (B = 0.220, 95%CI: 0.162-0.277 and p < 0.001); Table 4 ).

**Table 4:** Multiple Regression Analysis of Possible Factors Affecting Galanin and Glypican-4 Levels

	Coefficient of regression	95% Confidence Interval		t-statistic	p value
		Lower limit	Upper limit		
**Galanin**					
PCO	-0.472	-0.577	-0.368	-8.998	**<0.001**
BMI	0.113	0.097	0.129	14.170	**<0.001**
HOMA-IR	-0.007	-0.022	0.009	-0.863	0.391
HDL cholesterol	0.001	-0.002	0.005	0.714	0.477
Triglyceride	0.0005	-0.0004	0.001	1.106	0.272
LH	0.005	-0.001	0.011	1.650	0.103
mFG	-0.005	-0.023	0.012	-0.626	0.533
**Glypican**					
PCO	0.270	-0.128	0.667	1.350	0.181
BMI	0.220	0.162	0.277	7.565	**<0.001**
HOMA-IR	-0.016	-0.072	0.040	-0.556	0.580
HDL cholesterol	0.005	-0.008	0.017	0.722	0.472
Triglyceride	-0.001	-0.004	0.002	-0.895	0.373
fT	-0.134	-0.315	0.047	-1.475	0.144
FSH	0.029	-0.061	0.119	0.638	0.525
mFG	0.004	-0.062	0.070	0.122	0.903

## DISCUSSION

Our results show that patients with PCOS exhibited lower galanin and higher glypican-4 levels than controls. However, when the subjects were further divided based on the BMI, subgroup analyses showed that galanin levels were significantly lower in lean patients with PCOS as compared to the BMI-matched controls, whereas this difference did not reach significance in the overweight/obese group. As for glypican-4 levels, the overweight/obese PCOS group demonstrated higher levels than the controls, whereas lean groups yielded similar results. Both adipokine levels showed positive correlations with BMI as well as metabolic syndrome markers and androgenic profile; however, multivariate analyses demonstrated that PCOS and BMI appear to be independent risk factors affecting galanin levels, while only BMI was an independent risk factor affecting glypican-4 levels.

Galanin is involved in appetite, obesity, dyslipidaemia, insulin resistance and diabetes mellitus, hypertension, metabolic syndrome as well as reproduction ( 5 , 10 ). Numerous studies pointed out the relationship between galanin and metabolic syndrome. As a result, it can be used as a cardiovascular disease marker, demonstrating higher levels of this adipokine in diabetes mellitus, impaired glucose tolerance and gestational diabetes mellitus ( 22 – 24 ). Acar and cols. ( 25 ) reported that galanin levels were positively correlated with insulin resistance and triglycerides, also in obese children. Interestingly, physical activity is an effective stimulus to enhance galanin secretion ( 26 ). Fang and cols. ( 27 ) concluded that the galanin system is required for physical activity to relieve insulin resistance, causing a beneficial effect on exercise-induced GLUT-4 translocation. Galanin resistance, which is defined to be the discrepancy between high levels of circulating galanin and low glucose handling in the diabetic population, is the critical step in the development of type 2 diabetes mellitus ( 28 ). In contrast, galanin can increase insulin sensitivity, but the circulating levels of galanin are high in the diabetic group. Galanin resistance is thought to be highly related to obesity. However, there are very few studies about galanin levels in patients with PCOS. Baranowska and cols. ( 29 ) reported that galanin concentrations in PCOS were higher than those in the control group, but the difference was not significant statistically. Similarly, Bidzińska-Speichert and cols. ( 17 ) found that patients with PCOS, both obese (BMI ≥ 30) and non-obese (BMI < 30) had lower levels of galanin, similar to our data. The high galanin levels in the diabetic group suggest galanin resistance. However, the circulating levels were lower in PCOS patients, contrary to what was expected. Our results lead us to infer that there is a linkage between insulin resistance in PCOS disease and galanin deficiency. As galanin is an important hormone for elevating insulin sensitivity via GLUT-4 translocation ( 5 , 27 ), perhaps galanin deficiency may at least be one of the efficient factors responsible for insulin resistance in PCOS. Since galanin elevates insulin sensitivity via causing an increase in GLUT-4 translocation and a decrease in insulin secretion from the pancreas ( 5 ), the results of the present study made us hypothesise that galanin deficiency is associated with insulin resistance in the group of PCOS patients. Galanin resistance might not be valid in the PCOS group, unlike the diabetic group, presumably because of the young age of these women. However, as they get older and start to develop type 2 diabetes, galanin resistance arise, resulting in higher levels. Another reason might be that galanin resistance is also highly related to obesity; very few patients in the present study were obese, with a mean BMI of 25.2 kg/m^2^. Galanin is a target agent for sex steroids, serving as a molecular motifs integrating the control of metabolism as well as reproduction ( 9 ), and leading to an alteration in gonadotropin-releasing hormone (GnRH) secretion ( 30 ). It stimulates LH secretion in porcine, but inhibits it in the mice ( 11 , 12 ). Possibly, it interacts with LH in humans, such as in mice, so that LH levels could not be inhibited because of lower galanin levels in women with PCOS. Our results show that galanin levels are negatively correlated with LH levels.

Glypican-4 is also a newly identified adipokine, a cell surface proteoglycan, which interacts directly with the insulin receptor ( 15 ). Binding of glypican-4 to the insulin receptor regulates insulin activation and downstream signalling as an insulin sensitiser. *In-vitro* studies have found that when glypican-4 depletes, insulin receptor activation diminishes ( 15 ). Circulating levels are correlated with BMI and associated with insulin sensitivity ( 15 ). Recently, Ning and cols. ( 31 ) stated, for the first time, that serum glypican-4 levels were elevated in subjects with metabolic syndrome. Their data also showed a positive correlation with fasting blood glucose, fasting insulin and HOMA-IR in all subjects, in compliance with the data of the current study. Circulating glypican-4 levels are higher in subjects with impaired glucose tolerance and positively correlated with BMI, WHR and HOMA index ( 16 ). Leelalertlauw and cols. ( 32 ) also found elevated glypican-4 levels in obese children, increasing with higher degrees of obesity. Yoo and cols. ( 33 ) mentioned a gender-based difference in glypican-4 levels, demonstrating higher levels in men than in women. They declared a positive relationship between glypican-4 levels and WHR as well as the ratio of visceral to subcutaneous fat area. These authors also stated that glypican-4 levels correlated with cardiometabolic risk factors, including insulin resistance and arterial stiffness, and the measurements were independently associated with non-alcoholic fatty liver disease, in women. Nevertheless, there is only one pilot study evaluating glypican-4 levels in women with PCOS. Jędrzejuk and cols. ( 18 ) conducted a pilot study to determine the connection between glypican-4 and cardiovascular parameters in patients with PCOS. They concluded that glypican-4 levels were higher in women with PCOS than those of controls and correlated with cardiovascular risk factors, in particular fat distribution, in spite of the low mean BMI of 22 kg/m^2^ and the absence of lipid disorders in the evaluated subjects. Their results revealed a positive correlation with metabolic parameters, including insulin and HOMA-IR, as well as androgenic markers, similar to the present study. In our study, Multiple Linear Regression analyses showed that BMI was an independent risk factor for glypican-4 levels, and the significant effect of PCOS on glypican-4 levels in the univariate analysis disappeared. Little is known about the signalling functions of glypican-4; the role of glypican-4 in adipocytes and its relationship to metabolic regulation remains unknown. Since the intracellular post-receptor events after binding to the receptor are not exactly known, we think that further molecular studies should be done on this subject. Based on these data, it is assumed that, when insulin resistance is present in a subject, glypican-4 levels may increase to enhance insulin sensitivity, if glypican-4 is acting as an insulin sensitivity-enhancing agent. This way, glypican-4 agonists may be used as treating agents. Consequently, our data reveal that serum galanin levels are lower and glypican-4 levels are higher in women with PCOS as compared to controls. Further studies are needed to determine whether these adipokines could be used as additional markers for insulin sensitivity and lipid profile and whether they might play a role in the pathogenesis of PCOS, in which the risks of metabolic cardiovascular risks are increased. Multivariate analyses suggest that PCOS and BMI may be the best predictors affecting galanin levels with a decreasing and increasing effect, respectively; in turn, BMI was the best predictor affecting glypican-4 levels with an increasing effect. Our results suggest the use of innovative medical treatment options of insulin resistance in further studies. As, galanin deficiency might at least be one of the efficient factors associated with insulin resistance in PCOS, it is hypothesised that the administration of galanin can increase insulin sensitivity. Further research may also use glypican-4 agonists to relieve insulin resistance and androgen excess. Regarding the prospective design, a small sample size may result in limitations. In this study, it was not possible to subdivide the phenotypic characteristics in the patient group due to the small sample. Nevertheless, altered levels of these adipokines in relation with PCOS in even young and lean women suggest that the mechanisms related to these pathways require further studies.
